# Diagnostic significance of microRNAs as novel biomarkers for bladder cancer: a meta-analysis of ten articles

**DOI:** 10.1186/s12957-017-1201-9

**Published:** 2017-08-03

**Authors:** Hong-Bin Shi, Jia-Xing Yu, Jian-Xiu Yu, Zheng Feng, Chao Zhang, Guang-Yong Li, Rui-Ning Zhao, Xiao-Bo Yang

**Affiliations:** 1 Department of Urology, Ningxia People’s Hospital, No. 301 North Zhengyuan Street, Jinfeng District, Yinchuan, 750021 Ningxia China; 2 Ningxia Medical University, Yinchuan, 750004 Ningxia China; 3 Department of Urology, General Hospital of Ningxia Medical University, Yinchuan, 750004 Ningxia China

**Keywords:** Diagnosis, miRNA, Bladder cancer, Meta-analysis

## Abstract

**Background:**

Previous studies have revealed the importance of microRNAs’ (miRNAs) function as biomarkers in diagnosing human bladder cancer (BC). However, the results are discordant. Consequently, the possibility of miRNAs to be BC biomarkers was summarized in this meta-analysis.

**Methods:**

In this study, the relevant articles were systematically searched from CBM, PubMed, EMBASE, and Chinese National Knowledge Infrastructure (CNKI). The bivariate model was used to calculate the pooled diagnostic parameters and summary receiver operator characteristic (SROC) curve in this meta-analysis, thereby estimating the whole predictive performance. STATA software was used during the whole analysis.

**Results:**

Thirty-one studies from 10 articles, including 1556 cases and 1347 controls, were explored in this meta-analysis. In short, the pooled sensitivity, area under the SROC curve, specificity, positive likelihood ratio, diagnostic odds ratio, and negative likelihood ratio were 0.72 (95%CI 0.66–0.76), 0.80 (0.77–0.84), 0.76 (0.71–0.81), 3.0 (2.4–3.8), 8 (5.0–12.0), and 0.37 (0.30–0.46) respectively. Additionally, sub-group and meta-regression analyses revealed that there were significant differences between ethnicity, miRNA profiling, and specimen sub-groups. These results suggested that Asian population-based studies, multiple-miRNA profiling, and blood-based assays might yield a higher diagnostic accuracy than their counterparts.

**Conclusions:**

This meta-analysis demonstrated that miRNAs, particularly multiple miRNAs in the blood, might be novel, useful biomarkers with relatively high sensitivity and specificity and can be used for the diagnosis of BC. However, further prospective studies with more samples should be performed for further validation.

## Background

Bladder cancer (BC) as the second common urinary system malignancy is a leading cause of global cancer-related deaths. The incidences of BC were 37.5 per 100,000 in men and 9.3 per 100,000 in women [1]. Approximated 72,570 new cases of BC were diagnosed and resulted in 15,210 deaths in the USA in 2013 [1]. The 5-year survival rate after endoscopic resection is high as 80% because BC in majority of the cases is low grade and non-muscle-invasive [1–3]. However, 10–20% of non-muscle-invasive BC (NMIBC) will progress to muscle-invasive BC (MIBC), and in these cases, the survival rate dramatically drops to 56% [4, 5]. In addition, approximately 50–70% of NMIBC cases show cancer recurrence within the first 2 years of treatment, with up to 90% of MIBC cases showing recurrence overall [3, 6, 7]. Due to the high mortality (44%) related to MIBC and the frequent recurrence (70%) of cancer, early diagnosis and monitoring of disease progression is particularly important [4, 6].

At present, there are a number of conventional diagnostic methods for BC detection. These include cystoscopy, voided urine cytology, and detection of several urine-based markers such as NMP22 (nuclear matrix protein 22) and BTA (bladder tumor antigen). The most common way is cystoscopy coupled with voided urine cytology, which is highly sensitive [8, 9]. However, cystoscopy is expensive, invasive, and uncomfortable for patients, and requires an experienced technician, which hinders its widespread application in BC diagnosis [10]. Voided urine cytology only works well diagnosing BC in high grade and high stage, and thus hampers its utility in the detection of low-grade BC [11]. Currently used urine-based biomarkers NMP22 and BTA are simple, quick, and non-invasive methods for BC screening [12]. Unfortunately, these two biomarkers offer only low sensitivity and/or specificity detection. Hence, it is really urgent to find more sensitive but non-invasive biological markers to ensure the precise detection of early-stage BC.

miRNAs are a series of short (generally around 22 nucleotide long), single-stranded, and non-protein-coding RNA gene products that are vital for the regulation of gene expression. They work through binding their target mRNAs to cause degradation or translational silencing [13]. Recent evidence has suggested that abnormal miRNA profiles are related to the development, progression, and prognosis of various human cancers [14, 15]. miRNAs can present extensively in plasma, serum, and urine because they are protected from RNase degradation by some membrane-secreted vesicles and/or together with RNA-binding proteins [16]. Thus non-invasive biomarkers for the diagnosis of BC may be developed from the urine-based miRNAs and circulating miRNAs [17–24]. Unfortunately, the findings are inconclusive, which may be attributable to differences in sample size, ethnicity, miRNA profiling, and sample type. Therefore, a comprehensive analysis was administrated in this meta-analysis to further elucidate the diagnostic value of miRNAs in BC detection.

## Methods

### Documentation retrieval

The relevant literature in the ExcerptaMedica Database (EMBASE), PubMed, Chinese Biomedical Literature (CBM) database, and Chinese National Knowledge Infrastructure (CNKI) web database (updated until December 31, 2014) were searched using a combination of the terms “microRNAs” or “miRNA” or “miR”, “bladder cancer” or “bladder tumor” or “bladder urothelial cell carcinoma” and “sensitivity” or “specificity”, “ROC curve” or “diagnosis”. In order to retrieve the most relevant studies, manual filtration of the references was also conducted.

### Inclusion and exclusion criteria

The adopted literature should meet the following criteria: (a) the diagnostic value of miRNAs for BC detection must have been evaluated, (b) the authors must have used the gold standards (histopathological examinations), and (c) the studies must contain detailed information for constructing two-by-two tables, which comprise true positives (TP), true negatives (TN), false positives (FP), and false negatives (FN). And exclusion criteria are as follows: (a) abstract, review, comment, editorial, and case reports, (b) overlapping data, (c) studies investigating survival or prognosis of BC, (d) deficient data, even on asking the corresponding authors for more information, and (e) sample size <100. If more than one study used some common samples, only the most precise one with the most samples was selected.

### Data extraction and quality assessment

Data extraction from the selected publications was done using a standardized table by our two authors independently. And the extracted data contains first author’s surname, publication time, and country of origin, sample size, age, type of case, and control groups, sample specimen, studied miRNAs, detection method, TP, FP, FN, and TN, and information needed for quality assessment. For studies involving more than one type of miRNAs or sample specimen, the data were extracted, and the study on each miRNA was considered as an independent study. The quality of each article was evaluated by the revised quality assessment of diagnostic accuracy studies (QUADAS-2) checklists [25]. All disagreements about the collected data were adequately debated by investigators and arrived at a final consensus.

### Statistical analysis

For purpose of assessing the diagnostic value of miRNAs for BC, a bivariate meta-analysis was managed to obtain pooled negative likelihood ratio (NLR), positive likelihood ratio (PLR), sensitivity, diagnostic odds ratio (DOR), specificity, and corresponding 95% confidence intervals (CIs) [26]. Meanwhile, the bivariate summary receiver operator characteristic (SROC) curve was drawn and the area under it (AUC) was calculated to display the sensitivity and specificity of each study. The heterogeneity assumption between the studies were also measured through chi-square based Cochran’s *Q* and *I* ^2^ tests. A *P* value <0.10 and an *I* ^2^ value >50% were considered as significant heterogeneity [27, 28]. In order to examine the heterogeneity of origin, the sub-group analysis and meta-regression were performed according to the features (sample size, ethnicity, miRNA profiling, and specimen type). Finally, the potential publication bias of these literature was measured by Deeks’ funnel plot asymmetry test with the screening standard of *P* value <0.05 [29]. All *P* values were two sided and all statistical analyses in this meta-analysis were performed by using the STATA statistical software (version 11.0; Stata Corp, College Station, Texas, USA).

## Results

### Characteristics of eligible studies

**Fig. 1:**
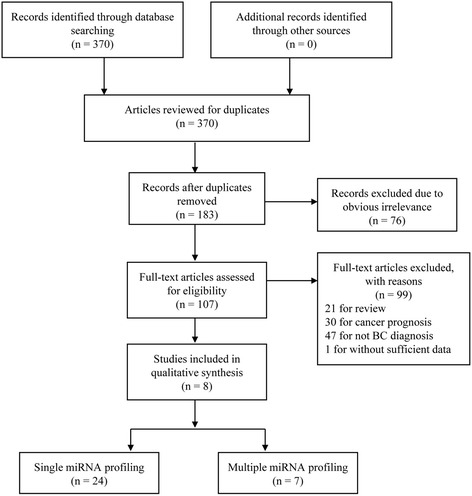
Flow chart of the study selection process

**Fig. 2:**
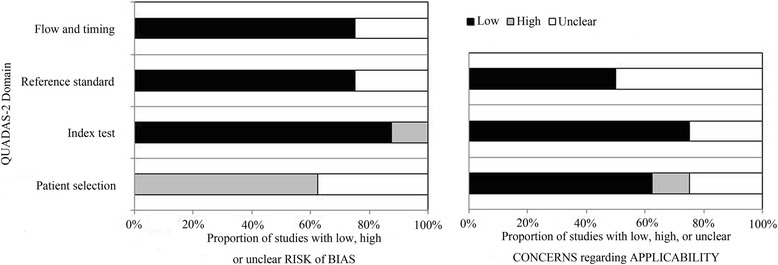
Overall quality assessment of included articles (QUADAS-2 tool)

There were 370 relevant articles picked out after preliminary screening in databases. The flow chart illustrates the study filtration procedures used for assessing the diagnostic potential of miRNAs in BC (Fig. 1). After a careful search and selection, eight publications meeting the final selection criteria stood out. Of these, the publication from Jiang XM et al. [18] studied two different populations (training and validation population) and one publication (Tölle A et al.) [19] researched two kinds of specimens (blood and urine); each population and specimen was considered as a separated article. Thus, in this meta-analysis there were 10 articles including 31 studies investigating 1556 cancer cases and 1347 controls in total. Furthermore, 24 of the 31 studies analyzed were focused on single-miRNA assays, the remaining seven articles were focused on multiple-miRNA assays. The main features of each research are shown in Table 1. All articles were published between 2012 and 2014. Four of the articles were performed in Asian populations and six of the articles were performed in Caucasian populations. The method of detecting miRNAs in all articles was the reverse transcription polymerase chain reaction (RT-qPCR). For miRNA detection, six articles were performed on urine samples and four were on blood samples. As shown in Table 1, all the studies analyzed in this article met the criteria for a moderate-high quality score. The risk of bias and applicability concerns graph for the included articles is presented in Fig. 2.

**Table 1:** Main characteristics of eight articles included in this meta-analysis

First author	Year	Country	Ethnicity	Sample size		Mean age		Patient spectrum	Source of control	Detecting method	MicroRNAs	Specimen	QUADAS
				Cases	Controls	Cases	Controls						
Zhou X	2014	China	Asian	112	78	65	62	Urothelial carcinoma of the bladder	Healthy individuals and patients with benign urological diseases	RT-qPCR	miR-106b	Urine	6
Zhang DZ	2014	China	Asian	50	21	75	65	Bladder urothelial cell carcinoma	Patients absence of malignancies and hematuria	RT-qPCR	miR-99a, miR-125b	Urine	4
Jiang XM	2014	China	Asian	120	120	63	63	Bladder cancer	Patients without bladder cancer	RT-qPCR	miR-152, miR-148b-3p, miR-3187-3p, miR-15b-5p, miR-27a-3p, miR-30a-5p	Serum	4
Jiang XM	2014	China	Asian	110	110	66	66	Bladder cancer	Patients without bladder cancer	RT-qPCR	miR-152, miR-148b-3p, miR-3187-3p, miR-15b-5p, miR-27a-3p, miR-30a-5p	Serum	4
Tölle A	2013	Germany	Caucasian	20	20	43	69	Bladder cancer	Healthy individuals and patients with benign urological diseases	RT-qPCR	miR-26b-5p, miR-144-5p, miR-374-5p	Blood	5
Tölle A	2013	Germany	Caucasian	20	19	43	69	Bladder cancer	Healthy individuals and patients with benign urological diseases	RT-qPCR	miR-520e, miR-618, miR-1255b-5p	Urine	5
Mengual L	2013	Spain	Caucasian	151	126	72	63	Bladder urothelial cell carcinoma	Controls without history of UCC	RT-qPCR	miR-25, miR-18a, miR-187 miR-204, miR-142-3p, miR-140-5p	Urine	3
Adam L	2013	USA	Caucasian	20	18	65	42	Bladder cancer	Patients without urologic malignancies	RT-qPCR	multiple miRNAs	Plasma	3
Miah S	2012	UK	Caucasian	68	53	72	62	Bladder urothelial cell carcinoma	Patients without history of UCC	RT-qPCR	miR-15a, miR-15b, miR-21, miR-23b, miR-24-1, miR-27b, miR-100, miR-133b, miR-135b, miR-183, miR-203, miR-211, miR-212, miR-328, miR-1224-3p	Urine	5
Snowdon J	2012	Canada	Caucasian	5	3	76	63	Bladder cancer	Healthy control patients without history of urothelial cancer	RT-qPCR	miR-125b, miR-126	Urine	5

QUADAS score ≥6 indicated high quality, QUADAS score 4–6 indicated moderate quality, QUADAS score ≤3 indicated low quality

*UCC* urothelial cell carcinoma, *RT-qPCR* quantitative reverse transcription polymerase chain reaction, *QUADAS* quality assessment of diagnostic accuracy studies

### Diagnostic accuracy of miRNAs for differentiating BC from controls

**Fig. 3:**
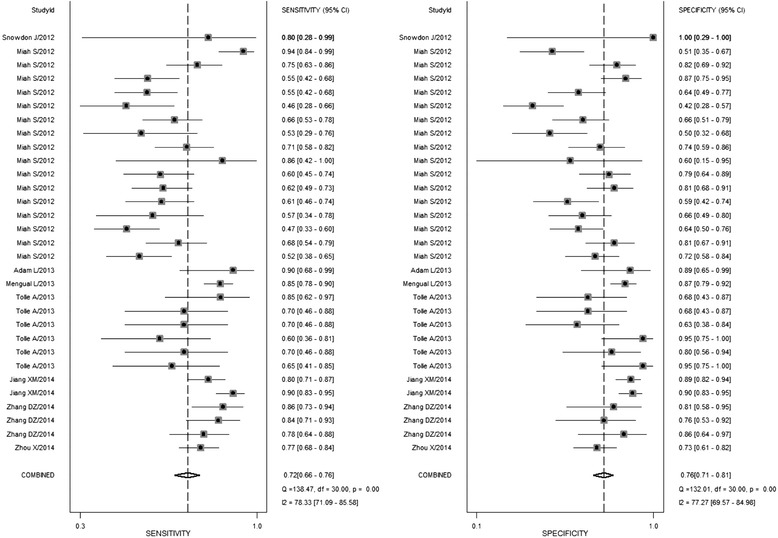
Forest plot of sensitivity and specificity of miRNAs for the diagnosis of BC

**Fig. 4:**
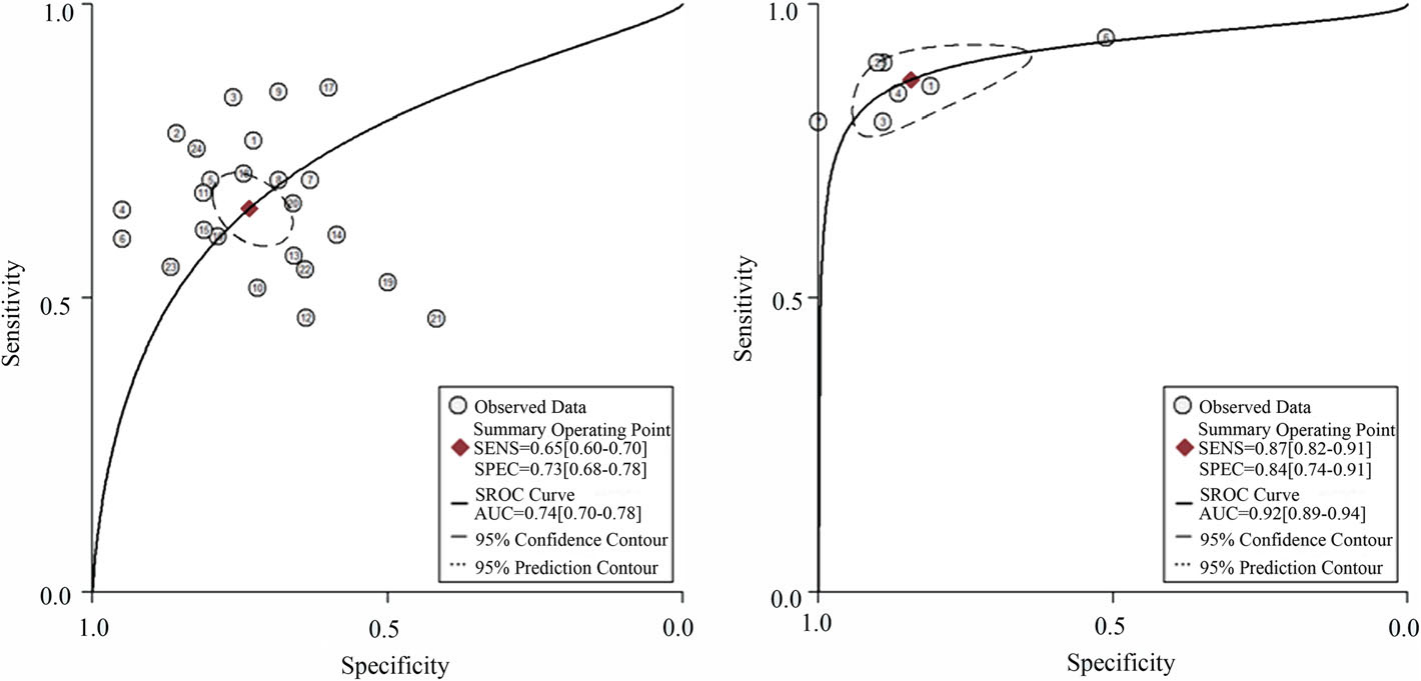
The SROC curve of miRNAs for the diagnosis of BC

The summarized estimates of the diagnostic accuracy of miRNAs that distinguish BC from controls are listed in Table 2. Thirty-one studies from 10 researches covering 38 types of miRNAs were analyzed. The pooled sensitivity and specificity of the miRNAs for the diagnosis of BC were 0.72 (95%CI 0.66–0.76) and 0.76 (95%CI 0.71–0.81), respectively (Fig. 3, Table 2). Besides, the pooled PLR, NLR, and DOR with 95%CIs for overall studies were 3.0 (2.4–3.8), 0.37 (0.30–0.46), and 8 (5.0–12.0), respectively. The SROC curve with an AUC of 0.80 (95%CI 0.77–0.84) displayed in Fig. 4 was for the overall study.

**Table 2:** Summary estimates of diagnostic criteria and their 95% confidence intervals

Sub-groups	Sensitivity	Specificity	Positive LR	Negative LR	DOR	AUC
	(95%CI)	(95%CI)	(95%CI)	(95%CI)	(95%CI)	(95%CI)
Ethnicity						
Asian	0.83 (0.78–0.87)	0.84 (0.77–0.89)	5.2 (3.5–7.8)	0.21 (0.15–0.28)	25 (13–48)	0.90 (0.87–0.92)
Caucasian	0.68 (0.61–0.73)	0.74 (0.68–0.79)	2.6 (2.0–3.3)	0.44 (0.36–0.54)	6 (4–9)	0.76 (0.73–0.80)
MiRNA profiling						
Single miRNAs	0.65 (0.60–0.70)	0.73 (0.68–0.78)	2.5 (2.0–3.1)	0.47 (0.40–0.56)	5 (4–8)	0.74 (0.70–0.78)
Multiple miRNAs	0.87 (0.82–0.91)	0.84 (0.74–0.91)	5.6 (3.3–9.3)	0.15 (0.11–0.20)	36 (22–61)	0.92 (0.89–0.84)
Sample types						
Blood-based	0.79 (0.68–0.87)	0.90 (0.85–0.93)	7.7 (5.2–11.3)	0.23 (0.15–0.37)	33 (16–65)	0.90 (0.87–0.93)
Urine-based	0.70 (0.64–0.75)	0.72 (0.67–0.77)	2.5 (2.0–3.1)	0.42 (0.34–0.53)	6 (4–9)	0.77 (0.73–0.80)
Sample size						
>40	0.70 (0.64–0.76)	0.75 (0.69–0.80)	2.8 (2.1–3.7)	0.40 (0.31–0.52)	7 (4–12)	0.79 (0.75–0.82)
≤40	0.74 (0.66–0.81)	0.81 (0.70–0.89)	3.9 (2.4–6.3)	0.32 (0.24–0.43)	12 (6–23)	0.80 (0.76–0.83)
Overall	0.72 (0.66–0.76)	0.76 (0.71–0.81)	3.0 (2.4–3.8)	0.37 (0.30–0.46)	8 (5–12)	0.80 (0.77–0.84)

*CI* confidence interval, *LR* likelihood ratio, *DOR* diagnostic odds ratio, *AUC* area under the curve

### Sub-group analyses and meta-regression

Sub-group analyses and meta-regression were conducted in order to reveal the inter-study heterogeneity attributed to ethnicity, miRNA profiling, specimens, and sample size (Table 2). The results suggested that the diagnostic accuracy of miRNA assays in Asian populations (SEN 0.83; SPE 0.84; PLR 5.2; NLR 0.21; DOR 25; AUC 0.90) was much better than that in Caucasian populations (SEN 0.68; SPE 0.74; PLR 2.6; NLR 0.44; DOR 6; AUC 0.76). The sub-groups based on miRNA profiling showed that multiple-miRNA assays were more accurate in detecting BC than single-miRNA assays, with a sensitivity of 0.87 vs. 0.65, specificity of 0.84 vs. 0.73, PLR of 5.6 vs. 2.5, NLR of 0.15 vs. 0.47, DOR of 36 vs. 5, and AUC of 0.92 vs. 0.74, respectively. Moreover, for studies on miRNA detection in the blood, the pooled sensitivity was 0.79, specificity was 0.90, PLR was 7.7, NLR was 0.23, DOR was 33, and AUC was 0.90. For studies using urine samples, the pooled sensitivity was 0.70, specificity was 0.72, PLR was 2.5, NLR was 0.42, DOR was 6.0, and AUC was 0.77. Thus, miRNA detection in the blood might have higher diagnostic accuracy than that in urine. However, the pooled estimates (SEN, SPE, PLR, NLR, DOR, and AUC) were close between studies conducted using large sample sizes (>40) with small sample sizes (≤40).

**Fig. 5:**
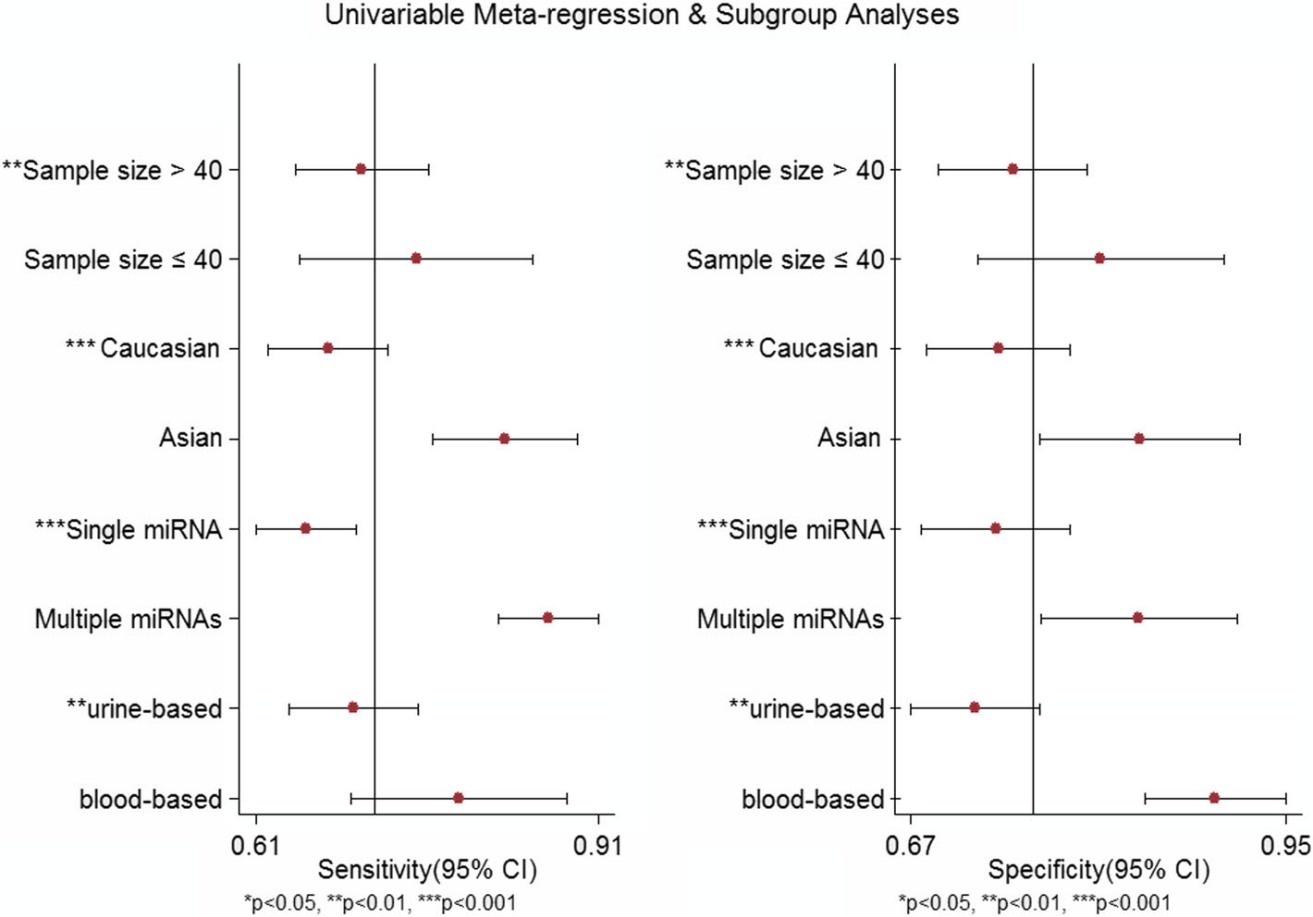
Univariable meta-regression for sensitivity and specificity of miRNAs for the diagnosis of BC

Furthermore, meta-regression analysis was also used to explore the potential sources of heterogeneity after adding eight pre-specified covariates (sample size, ethnicity, miRNA profiling, and specimen) to the bivariate model. The meta-regression analysis suggested that sample size (*P* value <0.01), ethnicity (*P* value <0.001), miRNA profiling (*P* value <0.001), and specimen (*P* value <0.01) could be the reasons of the heterogeneity in sensitivity and specificity between the studies (Fig. 5).

### Publication bias

**Fig. 6:**
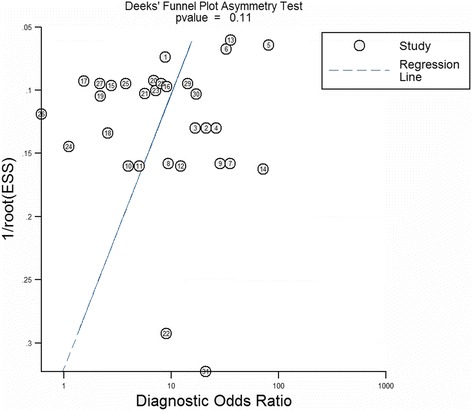
Deeks’ funnel plot asymmetry test of miRNAs for the diagnosis of BC

Deeks’ funnel plot asymmetry test was used to evaluate the possible publication bias in the included studies. Overall, the funnel plots showed symmetry and the slope coefficient was associated with a *P* value of 0.11 (Fig. 6). For the ethnicity, miRNA profiling, and specimen-based sub-group analysis, the slope coefficient showed *P* values >0.05 for all (not shown). All results confirmed that there was no obvious publication bias.

## Discussion

This meta-analysis is the first evidence-based analysis to assess the potential role of miRNAs for BC detection. This meta-analysis includes 31 studies from 10 researches covering 1556 cancer cases and 1347 controls in all, and the results indicate that miRNAs are potential novel and useful BC biomarkers with relatively high sensitivity and specificity that can be used for BC diagnosis.

Due to the high morbidity in men, high mortality for MIBC, and frequent recurrence, aggressive surveillance and timely intervention can dramatically improve the prognosis of BC. Currently, the method of the initial clinical diagnosis of BC—cystoscopy coupled with voided urine cytology—[8, 9] is expensive, invasive, uncomfortable for patients, and requires technical expertise. A meta-analysis including 36 studies with 14,260 cases was administrated to appraise the diagnostic value of cytology, indicating that the pooled specificity for the method was up to 96% (95%CI 94–98%). However, the pooled sensitivity was only about 44% (95%CI 38–51%) [30]. Several studies have also demonstrated that cytology has poor sensitivity for low-grade BC [31, 32]. Although urine-based NMP22 and BTA are also being used as simple, quick, and non-invasive methods for BC screening, their diagnostic accuracy is not satisfactory and shows high variability. One study analyzed the function of NMP22 in the BC diagnosis in 1331 participants and showed that the sensitivity was only 55.7% [33]. Other studies have reported sensitivities of NMP22 for BC diagnosis were ranging from 49.5 to 92.1%, whereas the specificities range from 66.0 to 87.3% [11]. Similarly, urinary BTA tests have diagnostic sensitivities ranging from 29 to 91% and specificities from 56 to 85% [11]. In comparison, miRNAs seem to have relatively high diagnostic accuracy with an overall pooled sensitivity of 0.72, specificity of 0.76, and an AUC of 0.80 in this meta-analysis.

It should be noted that available heterogeneity existed between these studies used in this paper; therefore, the effects of these confounding factors were explored via sub-group analyses. When stratified by miRNA profiling, the interesting finding was that single-miRNA assays showed relatively poor diagnostic performance with an AUC value of 0.74, while the diagnostic accuracy of multiple-miRNA assays is significantly higher with an AUC of 0.92. The results indicate that combination of multiple miRNAs might be more useful for the diagnosis of BC than single miRNAs. In addition, numerous studies have validated that single miRNAs can be used as accurate biomarkers for cancers. For example, Yang et al. performed a meta-analysis to evaluate the diagnostic performance of miRNA-21 in serum for lung carcinoma detection and suggested that miRNA-21 has diagnostic potential with an AUC value of 0.86 [34]. Another meta-analysis indicated miRNA-21 was a reliable biomarker for diagnosing breast cancer [35]. Wu et al. also proved miRNA-21 might be a potential biomarker for various early-stage cancers [36]. However, the diagnostic accuracy estimated in these meta-analysis studies is still lower than that of multiple-miRNA assays. In addition, no specific miRNA was reported to be a reliable candidate biomarker for BC. Furthermore, other meta-analysis studies that focused on various cancers have also indicated that multiple miRNAs were more promising as diagnostic biomarkers than single miRNAs. The reason could be that the occurrence and development of a severe malignancy involves complicated, multiple epigenetic and genomic abnormalities.

When stratified by sample types, the results indicate that comparing to urine-based assays, blood-based assays could be a better choice as a diagnostic tool. Ding et al. carried out a meta-analysis to evaluate the diagnostic value of miRNAs for pancreatic cancer and made a similar observation that blood-based assays were significantly better than non-blood-based assays [37]. However, Wei et al. performed a meta-analysis to measure the diagnostic value of miRNAs for cancers of the central nervous system and found that cerebrospinal fluid-based assays had more precise results than blood-based assays [38]. These results imply that different sample types may result in different diagnostic accuracies for miRNA detection. MiRNAs in the urine are derived from tumor cells, which slough off; therefore, they may detect only high-grade cancers possibly in the final stages. However, miRNAs in the blood are secreted from tumor cells by microvesicles, and different expression profiles can be detected in the initial stages of cancer. Thus, detecting miRNAs in the blood may have more diagnostic value than detecting the same in the urine.

Furthermore, the sub-group analysis by ethnicity suggests that the diagnostic accuracy of miRNA assays is significantly better in Asian populations than that in Caucasian populations, with a pooled DOR of 25.0 vs. 6.0 and AUC of 0.90 vs. 0.76, respectively. Li et al. conducted a meta-analysis to evaluate the diagnostic value of miRNAs for hematologic malignancies and found that Asian population-based miRNA tests yield an overall higher accuracy than tests in Caucasian populations [39]. However, other researchers did not observe any difference between Asian and Caucasian populations in their meta-analyses [40]. Moreover, Cui et al. obtained the opposite results indicating that miRNA assays might be more accurate in Caucasian populations when used for the diagnosis of breast cancer [41]. A potential explanation is that different ethnicities live in multiple environments and hold differing genetic backgrounds, lifestyles, and dietary habits, and therefore yield different miRNA expression profiles. The other potential explanation is that Caucasian populations come from Western developed countries, the medical services in Western developed countries are much better than developing countries in Asia, and they could accurately detect BC at an early stage; however, the change of miRNA expression profiles in early stage of tumor may not be obvious.

This study has several strengths notwithstanding a few limitations. This is the first meta-analysis to use as many as 1556 cancer cases and 1347 controls to summarize the diagnostic value of miRNAs in BC, which gives improved statistical power to the findings. The sub-group analysis confirmed that Asian population-based studies, multiple-miRNA profiling, and blood-based assays might yield increased diagnostic accuracy. Finally, this meta-regression analysis aimed to explore the sources of heterogeneity indicated that ethnicity, miRNA profiling, and sample type were independent confounding factors. However, the limitations of this study should also be acknowledged. Firstly, the cut-off parameters for miRNAs were different in the studies used and could be a potential source of heterogeneity. Secondly, this meta-analysis did not evaluate differences in the diagnostic accuracy of miRNAs presented in BCs with different clinicopathological features.

## Conclusions

This meta-analysis provides the first evidence of miRNAs being novel, useful biomarkers for the diagnosis of BC. Detection of miRNAs can be done with high sensitivity and specificity, particularly using multiple-miRNA blood-based assays. However, extensive functional evaluations and further population-based prospective studies with larger sample sizes and different ethnic groups are warranted to confirm and extend these findings.

## Abbreviations

BC: Bladder cancer
BTA: Bladder tumor antigen
CIs: Confidence intervals
DOR: Diagnostic odds ratio
FN: False negatives
FP: False positives
MIBC: Muscle-invasive BC
miRNAs: MicroRNAs
NLR: Negative likelihood ratio
NMIBC: Non-muscle-invasive BC
NMP22: Nuclear matrix protein 22
PLR: Positive likelihood ratio
SROC: Summary receiver operator characteristic
TN: True negatives
TP: True positives
